# Social and Demographic Factors Associated With Obese Children in the Age Group of 6-12 Years Attending a Tertiary Care Institute in Central India and the Prevalence of Depression in These Children: An Observational Study

**DOI:** 10.7759/cureus.41749

**Published:** 2023-07-11

**Authors:** Prakash J Mathew, Tushar B Jagzape, Anil Kumar Goel, Ajay Kumar, Tripty H Singh

**Affiliations:** 1 Pediatric Intensive Care Unit, Narayana Hrudayalaya, Bangalore, IND; 2 Pediatrics, All India Institute of Medical Sciences, Raipur, Raipur, IND; 3 Psychiatry, All India Institute of Medical Sciences, Raipur, Raipur, IND

**Keywords:** junk food, physical activity, screen time, depression, bmi, childhood obesity

## Abstract

Introduction: Childhood obesity in India is on the rise and is rarely raised as a concern. In the central Indian states, focus is largely on undernutrition. Thus, studies related to risk factors for being overweight and obese and the impact of obesity on the psychology of children are lacking. Hence, a hospital-based study with objectives to identify social and demographic factors associated with obesity and the estimation of the prevalence of depression among these children was conducted.

Methods: This observational study was conducted in a tertiary care institute in the state of Chhattisgarh from July 2020 to October 2021. Children with obesity as per the WHO (> 95^th^ percentile for the BMI) in the age group of 6-12 years were included after due consent. A proforma was administered targeting the objectives of the study and the Childhood Depression Rating Scale (CDR-S) and clinical evaluation identified the depressed.

Results: Among the 5,019 children screened during the study period, 54 met the inclusion criteria (1.07%). Fifty percent of children were from the upper middle class as per the Kuppuswamy scale. Seventy percent consumed junk food more than three times a week. Twenty-three children (42.6%) engaged in physical activity of > 1 hour and 49(90.7%) had a screen time of > 2 hours. The prevalence of depression among these children stood at 5.6% with the mean age being 11.67 years. Multiple logistic regression showed an inverse association of depression with physical activity.

Conclusion: Obesity is prevalent in higher socioeconomic groups. Many risk factors like screen time, junk food consumption, and physical activity are modifiable. The prevalence of depression increased with age. Physical activity showed an inverse relation to depression in obese children.

## Introduction

Childhood obesity is emerging as a significant issue in this century. In India, its prevalence has increased from 16.3% in 2001-2005 to 19.3% in 2010-2015 and is on the rise [1]. With undernutrition already a significant concern, obesity is likely to cause a double burden leading to economic impact in the form of increased expenditure for health care. Obesity may affect the working capacity of the individual. It also causes psychological problems like bullying, body shaming, and depression in obese individuals. In this way, economic and social stability is likely to be affected. But unlike undernutrition childhood increased weight and obesity are ignored due to lack of awareness in the general community and among doctors. The negligible number of studies is evidence for this neglect, especially in the central Indian population. A community-based study conducted in 2012 in selected schools of Bhilai, Chhattisgarh, placed the prevalence of obesity among adolescents at 8.4% but did not evaluate childhood obesity or factors associated with it [2]. Studies have shown association of depression with obesity among the children of the Western population [3], but scarce data exist on any such association among Indian children. Hence, a hospital-based study was designed to evaluate certain social and demographic factors like socioeconomic status, family history of obesity, frequency of junk food consumption, exercise duration, and screen time in association with obesity and also assess depression in obese children using the Child Depression Rating Scale (CDR-S) and clinical evaluation by a psychiatrist wherever indicated.

## Materials and methods

Methods

Ethical clearance was obtained from the institute ethics committee of All India Institute of Medical Sciences (AIIMS) Raipur, vid letter no 1042/IEC-AIIMSRPR/2020 from IEC AIIMS Raipur. IEC Proposal no: AIIMSRPR/IEC/2020/512 Chhattisgarh. The study design was a hospital-based cross-sectional study conducted at the Department of Pediatrics AIIMS Raipur from July 2020 to October 2021.

Study participants

All children in the age group of 6-12 years with BMI > 95th percentile as per the World Health Organization reference charts were included. Those with intellectual disability (ID) or developmental delay who could not answer the preformed proforma or the CDR-S questionnaire, or having an iatrogenic cause for obesity were excluded. A total of 5,019 children in the age group of 6- 12 years visited AIIMS Raipur pediatric department during the study duration out of which 142 were obese. But 86 were excluded as they were on steroids (iatrogenic cause), and one was excluded for speech delay and one for profound hearing loss along with ID. So, in the end, 54 out of 142 met the inclusion criteria.

Data collection tools

A proforma noting the basic demographic details, and factors under evaluation like socio-economic status, family history of obesity, frequency of junk food consumption, exercise duration, and screen time in association with obesity was used along with the Child Depression Rating Scale (CDR-S revised) to assess depression.

Data collection

All children visiting the Department of Pediatrics underwent anthropometric evaluation. Children identified to be obese according to the WHO BMI criteria, after informed consent, were given the proforma for demographic details, and factors associated with obesity were studied. Subsequently, the child was administered the CDR-S revised scale for assessing depression. A cut-off score of 28 was kept above which the child was referred to a psychiatrist for confirmation of depression. The child was termed to be clinically depressed only after psychiatric confirmation.

Statistical analysis

Data were entered into Microsoft Excel, and using IBM SPSS Statistics for Windows, Version 27 (Released 2020; IBM Corp., Armonk, New York, United States), data were analyzed. Confidence intervals were set at 95%. The Shapiro-Wilk test was used to assess the normalcy of the distribution, and Fisher’s exact test and Chi-square test were used to interpret statistical significance of normally distributed data. The Kruskal Wallis test was used to test the significance of categorical non-normally distributed data and the Mann-Whitney U test for non-normally distributed continuous variables. 

## Results

In the age group of 6-12 years, 5019 children visited the OPD during the study duration. Among them, 142 were found to be obese, out of which 54 met the inclusion criteria. Basic demographic details are given in Table 1.

**Table 1:** Basic demographic variables of study participants IQR: Interquartile range; BMI: body mass index

Demographic variables	Mean ± SD	Median (IQR)	Min-Max	Frequency (%)
Age (Years)	9.11 ± 2.02	9.00(7.25-11.00)	6.00-12.00	
Gender				
Male				37(68.5%)
Female				17(31%)
Socioeconomic status (Modified Kuppuswamy scale)				
Upper Middle				27(50.0%)
Lower Middle				21(38.9%)
Upper Lower				3(5.6%)
Lower				3(5.6%)
BMI (Kg/m^2^)	24.61 ±4.15	23.74(21.63-26.00)	19.10-40.10	

Fifty percent of obese children were from the upper middle socioeconomic status while only about 5% were from the lower socioeconomic status as per the modified Kuppuswamy scale. Family history of obesity among the first-degree relatives of obese children was found to be 64.8% (95% CI 50.6-77.0%); 70.4% (95% CI 56.2-81.6) of obese children had consumption of junk food greater than 3 per week, and junk food consumption was also more prevalent in the upper-middle-class population (Fisher’s exact test X2 = 13.8, p-value 0.011). In this study, we used the standard definition of junk food as "foods (packed or non-packed, processed or non-processed) that contain little or limited presence of proteins, vitamins, phytochemicals, minerals, and dietary fiber but are rich in fat (saturated fatty acids), salt, and sugar and high in energy (calories) that are known to have negative impact on health if consumed regularly or in high amounts". In common language, we considered junk food as packed food, pizza, pasta, noodles, samosa, chips, kurkure, etc. The recommended daily exercise duration of greater than one hour was present only in 42.6% (95% CI 29.5- 56.7%) of obese children. The operational definition of exercise used in this study was any bodily movement produced by skeletal muscles which requires expenditure of energy with the target of remaining active. This included structured cardiovascular exercises in children for example swimming as well as leisure activities like playing games and also but not limited to chores like cycling or walking to school. The particular activity needs to be strenuous enough to cause sweating. An alarming 90.7% (95% CI 78.9% - 96.5%) had a screen time of more than two hours per day, and this was also found to be more for the upper-middle-class population (Fisher’s exact test X2 = 16.1, p-value 0.004) (Table 2).

**Table 2:** Factors under study and their frequency in obese children CDRS: Child Depression Rating Score; IQR: interquartile range

Variables	Mean ± SD	Median (IQR)	Min - Max	Frequency (%)
Positive family history of obesity				35 (64.8%)
Degree of relation to an obese family member (1st)				37 (100%)
Junk food consumption				
< 1 time/week				5 (9.3%)
1-3 times /week				11 (20.4%)
>3 times/week				38 (70.4%)
Exercise				
< 1 hour/day				31 (57.4%)
>1 hour/day				23 (42.6%)
Screen time				
< 2 hours/ day				5 (9.3%)
>2 hours/ day				49 (90.7%)
CDRS score	20.13 ± 3.66	19.00 (18.00-21.00)	17.00 -35.00	
Clinical depression present				3 (5.6%)

The prevalence of depression in these children evaluated using the CDRS scale and confirmed by clinical assessment by a psychiatrist was found to be 5.6% (95% CI 1.4%- 16.3%) of the obese children. Depression was found to be more prevalent in the older age groups than younger with the mean age being 11.6 among the obese and depressed children vs 8.9 years for the non-depressed obese population (Wilcoxon-Mann-Whitney U test W= 136.5; p-value 0.023). No gender difference was noted among the depressed population (p-value 0.230) (Table 3).

**Table 3:** Mean age of depressed obese participants v/s non-depressed counterpart IQR: Interquartile range

Age (Years)	Clinical Depression		Wilcoxon-Mann-Whitney U Test	
	Present	Absent	W	p value
Mean (SD)	11.67 (0.58)	8.96 (1.97)	136.500	0.023
Median (IQR)	12 (11.5-12)	9 (7-10)		
Range	11 - 12	6 - 12		

A multiple logistic regression analysis conducted for factors associated with depression showed inverse significance of depression with physical activity.

## Discussion

Obesity is a form of malnutrition, defined by the WHO as BMI of more than 2SD for age and sex for children, more than five years of age [4]. It is caused by excess calorie accumulation in the body. The prevalence is on the rise across the world and in India too, with studies indicating prevalence as high as 19.3% [1]. Community-based studies on obesity in central India are scarce, and one such study was conducted in Bhilai, Chhattisgarh projecting an 8.4% prevalence of obesity [2]. Hospital-based studies are few in India and none for the central Indian population, hence the importance of this study.

Meta-analysis on the prevalence of obesity in different age groups shows obesity to be more prevalent in adolescents than children in India [5]. Our study also reflects such a trend. In India, obesity is still a rich man’s disease as is reflected in our study with 50% of obese belonging to the upper middle class and only 5.6% belonging to the lower socioeconomic group. This contradicts the Western studies which have documented a higher prevalence of obesity in the lower socioeconomic strata [6] but are congruent with Indian studies that portray a similar picture [7].

The cause is multifactorial and comprises genetic, social, environmental, and lifestyle factors. Family history of obesity reflects both genetic predisposition and common family diet and lifestyle factors that influence obesity. Our study showed 64.8% of obese children having a first-degree relative who is obese and further studies are required to prove correlation. A similar pattern was found in a Ukrainian study that found positive correlation between the BMI of children and their mothers [8], and also in twin studies which showed 77% inheritance of obesity-associated genes [9,10].

Managing lifestyle factors is the most effective control strategy at an individual and family level. An accumulation of calories can be caused by increased intake, decreased utilization, or both. Hence, diet is an obvious factor for the control of obesity. Our study estimated the frequency of junk food consumption per week, and it was found to be 70% (>3 times per week) in obese children. This is similar to trends in the Western population [11,12]. This was highest in the higher socioeconomic strata (88.9% of upper middle class) indicating a misuse of the higher purchasing power of the affluent population towards faulty food habits than healthy alternatives. Studies from other parts of India also support such a trend [13].

Exercise burns out calories. Recommendations state that children should be engaged in active play for at least a duration of one hour per day. 57.4% did not follow this recommendation. Similar trends were found throughout the world in obese populations, where a rise in obesity was noted in populations with the least physical activity [14,15]. A factor promoting a sedentary lifestyle was postulated to be screen time. Recommended screen time for the age group is less than two hours per day [16]. But a whopping 90.7% of obese children had a screen time longer than this in our study. This has been a major contributor to obesity around the globe and also in India [17]. A similar study conducted in Tamil Nadu showed excess screen time in only 52.5% of obese children. This rise in screen time trends is consistent with Western data that documented a progressive increase in screen time with age and technological advancements [18]. This dramatic rise may also be attributed to the COVID-19 pandemic and associated restrictions though not statistically evaluated in this study. Like junk food, screen time was noted to be higher in the higher socioeconomic category.

The relation of psychiatric illnesses to obesity has been studied in the Western population but rarely in India. Associated psychiatric and behavioral disorders include anxiety, attention deficit hyperactivity disorder, and depression [3,19]. Depression in obese was found to be significant with causes as simple as teasing to complex psycho-socio-hormonal interactions. An overall prevalence of clinical depression in obese was noted to be 5.6% in our study with no female predisposition as indicated in others [20]. The average age of the depressed children (11.6 years) was higher than that in the non-depressed obese population (8.9 years) with significant statistic correlation of depression with age. Hence, older children and adolescents tend to be more depressed than younger children. No national reference could be found for comparison. A logistic regression analysis of the significant variables under study was conducted which showed inverse association of depression with physical activity. It indicated that physically active but obese children are less likely to suffer from depression. The results of our study may not be generalizable as the major limitation of this study was it was a single-center study. It does not reflect the exact seriousness of the situation. Moreover, the study had to be conducted during the COVID-19 pandemic when non-serious ailments were discouraged to attend the hospital. This severely restricted participation. This study is an observational study with no comparison group; hence, associations and correlations could not be statistically proved. A larger multicentric observational study with a larger sample size and comparison group is required to confirm findings in this study.

## Conclusions

The prevalence of obesity in children in the age group of 6 to 12 years in a clinical setting was found to be 1.07%. Lower values than that found in the community may indicate poor awareness of childhood obesity as a disease and the need for intervention. Obesity in first-degree family relatives, lifestyle factors like junk food consumption, low physical activity, and increased screen time are proposed to be possible factors identified in causing obesity. Higher prevalence of obesity in children of higher socioeconomic status shows misuse of resources in promoting an unhealthy lifestyle. This again may be due to the lack of awareness. Depression in obese children was estimated at 5.6% with an inverse relation to physical activity but requires further studies for discovery of more associated and causative factors.
